# The Honey Bee Body Surface as a Microbial Hub: Connectivity Shaped by Monoculture vs. Polyculture Farming

**DOI:** 10.3390/insects17010053

**Published:** 2026-01-01

**Authors:** Baobei Guo, Xueyan Yi, Qihang Sun, Ke Sun, Lina Guo, Yuan Guo

**Affiliations:** 1 Pomology Institute, Shanxi Agricultural University, Taiyuan 030031, China; 2 Shanxi Key Laboratory of Fruit Germplasm Innovation and Utilization, Taiyuan 030031, China; 3 College of Animal Science, Shanxi Agricultural University, Jinzhong 030801, China; 18783584670@163.com (X.Y.); qihangsun1999@163.com (Q.S.); 13239896557@163.com (K.S.); linaguo@126.com (L.G.); 4 College of Horticulture, Shanxi Agricultural University, Taiyuan 030031, China

**Keywords:** bee microbiome, agricultural intensification, microbial source tracking, co-occurrence networks, ecological stability

## Abstract

Honey bees, as vital pollinators and essential contributors to terrestrial ecosystems, play a critical role in maintaining biodiversity and ecological stability. Beyond their role as pollinators, honey bees are increasingly recognized as bioindicators of environmental health, with their microbiomes reflecting habitat quality, agricultural practices, and broader ecological conditions. This study examines the impact of monoculture and polyculture systems on bee-associated microbiomes, focusing on microbial diversity, composition, and functional roles. Microbial communities from floral surfaces, pollen, nectar, foraging bees, hive matrices, and bioaerosols were analyzed across three agricultural plots: a rape monoculture, a pear monoculture, and a polyculture plot. Using 16S rRNA amplicon sequencing, network co-occurrence analysis, and microbial source tracking, the findings reveal that plant species and cultivation methods significantly shape microbial dynamics (Adonis = 0.67 ***). Floral microbiomes exhibit host specificity (Adonis = 0.73 ***), while the honey bee body surface functions as a microbial hub linking environmental, floral, and hive microbial networks (average degree pear: 21.86; rape: 21.96). The polyculture system improves microbial diversity due to the diversity of nectar plants, enhancing ecosystem connectivity and potentially benefiting honey bee health. These results highlight the ecological importance of optimizing agricultural practices to preserve microbial diversity, enhance honey bee health, and maintain ecological stability.

## Simple Summary

Honey bees are not only important pollinators but also bioindicator species, one key factor being that their microbial communities reflect habitat quality and agricultural practices. This study explored how growing single crops (monoculture) versus multiple crops (polyculture) affects the microbial diversity and networks associated with honey bees. Researchers analyzed microbial communities from flowers, pollen, nectar, honey bees, and hives across three types of farms: a rape monoculture, a pear monoculture, and a polyculture system. We found that monoculture farming reduces microbial diversity, which can negatively affect honey bee health, while polyculture farming supports diverse and interconnected microbial communities that benefit both honey bees and the environment. These findings emphasize the need for sustainable farming practices to protect honey bee health, maintain biodiversity, and support ecological and agricultural stability.

## 1. Introduction

As vital pollinators and vital contributors to the productivity and structure of terrestrial ecosystems, honey bees (*Apis mellifera*) are crucial to maintaining biodiversity and ecosystem stability, supporting the livelihoods of farmers and beekeepers, and guaranteeing global food security [1,2]. Apart from their role as pollinators, honey bees are also important biological indicators of environmental health, as their well-being is closely linked to factors such as habitat quality, pesticide exposure, and agricultural practices [3,4,5]. Despite their significance, the broader ecological roles of honey bees remain profoundly underestimated [6,7]. In recent years, the health of honey bee populations has faced significant challenges due to habitat fragmentation, monoculture expansion, climate change, and the overuse of agrochemicals, all of which have led to population declines and disrupted ecosystem services [4,8].

A growing body of research highlights the crucial role of the bee microbiome—the diverse community of microorganisms inhabiting their body and surroundings—in shaping their health, immunity, and overall resilience [9,10,11]. These microbial communities influence key physiological processes, including digestion, pathogen defense, and detoxification, enabling bees to adapt to their ever-changing environments [12,13,14]. Consequently, elucidating the composition, diversity, and functional attributes of bee-associated microbiomes constitutes a critical avenue for mitigating the adverse effects of agricultural intensification on pollinator health and resilience. Insights derived from such investigations can directly inform evidence-based conservation strategies, guide the development of sustainable agricultural practices, and safeguard the continuity of pollination services essential to global agro-ecosystem stability [10,11,15,16].

Agricultural intensification has led to significant shifts in planting methods, broadly categorised into monoculture systems and diversified planting models. Monoculture, characterized by the cultivation of a single crop species over vast areas, is often favored for its economic efficiency and simplified management. Resource homogeneity, soil depletion, and increased susceptibility to pests and diseases can result from it, which necessitates the heavy use of chemical inputs like fertilizers and pesticides [17]. Planting regime—monoculture versus diversification—shapes the bee microbiome both within hosts and in their immediate habitat, yet this mechanism remains a critical blind spot in pollinator research. Because these symbionts govern immunity, nutrition, and detoxification, quantifying how cropping systems restructure their composition, diversity, and function is essential to translate farm-level management into pollinator resilience [18,19]. However, systematic evidence linking specific agricultural practices and planting regimes to precise alterations in the honey bee microbiome remains scarce. This gap obstructs the development of evidence-based farming strategies that safeguard pollinator health. We close it by dissecting the microbial response to contrasting planting models, supplying the missing mechanistic connection between agricultural intensification and honey bee health.

This study aims to investigate how planting practices influence bee microbiomes by comparing microbial communities in monoculture and polyculture agricultural systems. We specifically seek to address the following research questions: (1) How do monoculture and polyculture systems differ in shaping the composition and diversity of bee-associated microbial communities? and (2) What are the functional roles of these microbial communities in supporting bee health and ecosystem stability? This research hypothesizes that polyculture systems enhance microbial diversity and functional stability, while monocultures reduce diversity and may impair key physiological functions. Our findings emphasize the need for sustainable practices to preserve microbial diversity and support ecological stability in agricultural ecosystems.

## 2. Materials and Methods

### 2.1. Experimental Design and Treatments

The experimental design comprised three distinct sampling plots: (1) a rape monoculture plot (35.18881° N, 110.89236° E, 0.6 ha) demonstrating exclusive floral dominance (≥95% coverage), buffered by a 0.5 km radius barrier zone devoid of competing nectariferous species; (2) a pear dominated orchard (35.151852° N, 110.941844° E, 1.0 ha) maintaining ≥ 95% coverage; (3) a polyculture plot (35.168372° N, 110.835728° E) integrating sequential bloomers including pear (50%), rape (25%), peach (*Prunus persica*, 10%), *Orychophragmus violaceus* (9%), dandelion (*Taraxacum officinale*, 3%) and supplementary nectar-providing taxa (3%) based on phenological phase monitoring. Monoculture and polyculture plots were managed uniformly, with no pesticides sprayed 15 days before and during honey bee pollination. Irrigation and fertilization practices were consistent across all plots to ensure comparability. Specifies no interference or management practices applied during the experimental period. All three plots were located in a flat agricultural landscape, with consistent surrounding environments and no villages or other disturbance factors.

Before the experiment, 20 honey bee (*Apis mellifera*) colonies were managed under standard beekeeping protocols by the same apiarist in Yuncheng City, Shanxi Province, China (Figure S1). Fifteen colonies were selected for experimental use. Equalization procedures were conducted on 25 and 26 March 2022 during suboptimal foraging conditions to ensure maximal retention of foragers within hives, enabling accurate worker population assessment. Each colony was standardized to contain one mated queen; two brood frames with eggs, larvae, and capped pupae; three frames covered with adult worker bees; one honey frame; and one pollen frame containing approximately half to one full frame of stored pollen, plus two empty wax foundation combs, all housed in a standard Langstroth hive. Visual inspections confirmed the absence of clinical signs of major brood diseases or parasitic infestations. Subsequently, five colonies were placed in each experimental plot. Samples were collected after a standard 3-day acclimatization period to minimize novel environment-induced behavioral artifacts.

### 2.2. Sample Collection

Microbial samples were collected on 29 March 2022 following three consecutive days of normal honey bee foraging activity. Floral microbial communities, pollen, nectar, foraging bees, hive matrices (stored pollen and honey), and environmental bioaerosols were sampled across pear monoculture, rape monoculture, and polyculture plots. For each matrix, five biological replicates were obtained.

Floral microbiomes were collected from fully opened blossoms, while pollen, bee body, and nectar were obtained through standardized PBS-based elution or direct aspiration. Foraging bees were subjected to surface decontamination before crop dissection to isolate internal microbiomes. Hive matrices were cored from central combs under sterile conditions. Environmental bioaerosols were collected using elevated filter traps. Rape monoculture and polyculture plots were sampled for respective floral taxa (including pear, rape, peach, *Orychophragmus violaceus*, and dandelion) and bee-associated microbiomes. Due to the structural characteristics of northern rape flowers, rape nectar was not collected. In polyculture plots, only pear nectar was manually collected, while other nectar plants were sampled for floral microbiomes exclusively. All specimens were cryopreserved in liquid nitrogen and promptly stored in a −80 °C freezer.

A complete description of sampling procedures, washing steps, elution protocols, and plant-specific sampling adaptations is provided in Supplementary Methods.

### 2.3. DNA Extraction and Bioinformatic Analysis

Microbial DNA from all sample types was extracted using the FastDNA™ Spin Kit (MP Biomedicals, Irvine, CA, USA). DNA integrity was verified by spectrophotometry and agarose gel electrophoresis. The V3–V4 region of the bacterial 16S rRNA gene was amplified using primers 338 F and 806 R, and the resulting amplicons were purified, quantified, and sequenced on an Illumina MiSeq PE250 platform (San Diego, CA, USA).

Raw reads were processed using a standard amplicon workflow, including quality filtering (fastp), merging (FLASH), and denoising (DADA2) to obtain ASVs. Taxonomic classification was performed using Qiime2 (v2023.7) with the SILVA v138 database, followed by rRNA operon copy number normalization using rnDB (v5.8).

Detailed PCR conditions, reagent parameters, and bioinformatic scripts are provided in the Supplementary Materials.

### 2.4. Statistical Analysis

To analyze the microbial community differences, the ‘adonis’ function from the ‘vegan’ package in R (v4.0.3 available at https://CRAN.R-project.org/package=vegan, accessed on 22 February 2024 [20]) was utilized to perform permutational multivariate analysis of variance (PERMANOVA) or nested PERMANOVA (*p* < 0.001). This analysis was based on Bray–Curtis distances, which were computed to quantify bacterial beta diversity. The diversity was subsequently visualized using nonmetric multidimensional scaling (NMDS). To investigate the complexity and interaction patterns of microbial communities across different farming systems, we analyzed the topological features of microbial co-occurrence networks. Network co-occurrence analysis was carried out using the Fast UniFrac method to construct microbial co-occurrence networks based on Bray–Curtis distance. The network properties, including average degree, modularity, and network cohesion, were evaluated using Gephi (v0.9.2, https://gephi.org/, accessed on 27 September 2024) [21]. In addition, R packages such as ‘psych’ and ‘reshape2’ were employed for network analysis, and the resulting networks were visualized in Gephi. Additionally, microbial source tracking is performed using the FEAST (v0.1.0 Fast Expectation-Maximization for Microbial Source Tracking) tool [22,23]. This method reconstructs and quantifies the origin of microbes within the bee microbiome based on known environmental samples, thereby assessing the impact of different farming practices on bee-associated microbial communities. All statistical analyses are conducted in R (v4.0.3) for data visualization and result generation, ensuring reproducibility and clarity of the analysis outcomes.

## 3. Results

### 3.1. Plant-Driven Differentiation of Floral and Bee-Associated Microbiomes

**Figure 1:**
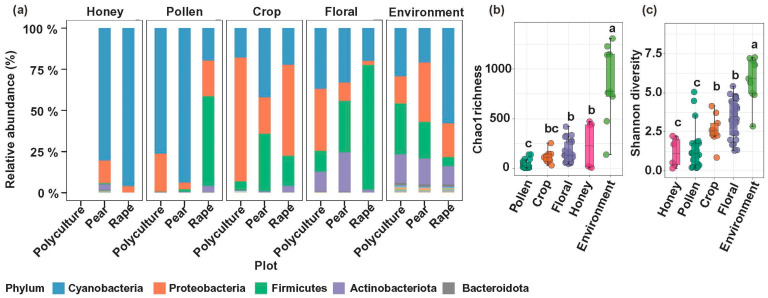
Comparative analysis of microbial assemblages across foraging-associated niches in the polyculture plot, pear monoculture plot, and rape monoculture plot. (**a**) Relative abundance of the dominant bacterial phyla in Honey (honeycomb-stored honey), Pollen (bee bread), Crop, Floral (polyculture samples from pear, rape, peach, *Orychophragmus violaceus*, and dandelion flowers; monoculture samples from pear and rape flowers), and Environment samples. The top five most abundant phyla were percent. (**b**) Chao1 richness and (**c**) Shannon diversity indices among these niches. Different letters (a–c) indicate significant differences (*p* < 0.05).

We present a comparative analysis of bacterial diversity across trophic levels within the honeybee pollination network. Despite repeated efforts, microbial DNA extraction failed in all comb samples (stored honey and pollen) from the polyculture plot. In contrast, Cyanobacteria predominated within honey extracted from comb bacterial communities across all successfully analyzed samples from both monoculture plots (pear and rape). Except for rape plots, bacterial communities were predominantly composed of Cyanobacteria and Proteobacteria in the pollen samples. The crop microbiota was characterized by three dominant phyla (Cyanobacteria, Proteobacteria, and Firmicutes), with particularly high relative abundance of Proteobacteria (Figure 1a). Environmental samples displayed the highest bacterial diversity and abundance. A progressive decline was observed along the gradient from the environment to floral surfaces, crops, pollen, and stored honey (Figure 1b,c).

**Figure 2:**
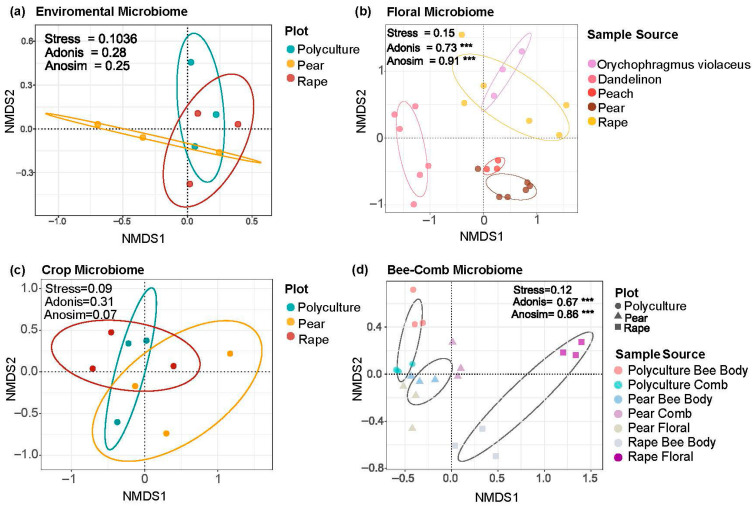
Non-metric multidimensional scaling (NMDS) visualization of microbial community structure across various sample types and cultivation plots. The significant variation in floral microbiomes highlights the crucial influence of plant species, cropping systems, and agricultural practices on microbial community structure, including distinct clustering observed in pear and rape monoculture plots within the honeybee pollination system. (**a**) Environmental microbiome samples exhibit a dispersed distribution with no distinct clustering patterns among the polyculture, pear monoculture, and rape monoculture plots. Ellipses represent the 95% confidence intervals of sample groupings based on microbial community composition. (**b**) Floral microbiomes exhibit significant variation among plant species (Adonis = 0.73 ***). (**c**) NMDS plot of honey crop microbiomes from foraging bees, which did not form clear clusters across different cultivation plots (Adonis = 0.31). (**d**) Bee-associated microbial communities (comb and body surface), demonstrating that bee body microbiomes are significantly influenced by the type of cultivated plants (Adonis = 0.67 ***). Significance is as follows: *** *p* < 0.001.

Environmental samples showed dispersed community structure but similar alpha diversity across sampling sites (Figure 2a and Figure S2a). In contrast, floral microbiomes from different cultivation plots formed distinct clusters. The composition of floral microbiomes demonstrated significant variation among plant species (Adonis = 0.73 ***, Figure 2b), with the monoculture plots of pear and rape exhibiting notably distinct distributions compared to the polyculture plot (Adonis = 0.67 ***, Figure 2d). This observation is consistent with findings in the floral microbiomes of peach and rape, which exhibited significantly higher diversity and richness compared to those of other plant species (Figure S2b). The Venn diagram reveals that the number of shared ASVs between different plant species is relatively small, while each plant species harbors a considerable number of unique ASVs (Figure S3). This indicates that the composition of floral microbiomes is highly specific to each plant species. Only a limited number of core microbial taxa are capable of coexisting across the floral niches of multiple plant species.

Bee-associated niches exhibited weaker plant-driven differentiation. Crop microbiomes showed neither clear clustering nor significant variation in alpha diversity across cultivation plots (Adonis = 0.31; Figure 2c and Figure S2c). However, hive-associated communities and bee surface microbiota were more sensitive to cultivation type, forming distinct clusters in monoculture plots and showing higher diversity in polyculture and rape plots (Figure 2d and Figure S2d). These patterns indicate that while floral microbiomes are strongly plant-specific, bee-associated microbiomes display variable responsiveness to cultivation practices depending on the niche.

### 3.2. Bee Body as a Microbial Hub with Distinct Responses to Cultivation Plots

**Figure 3:**
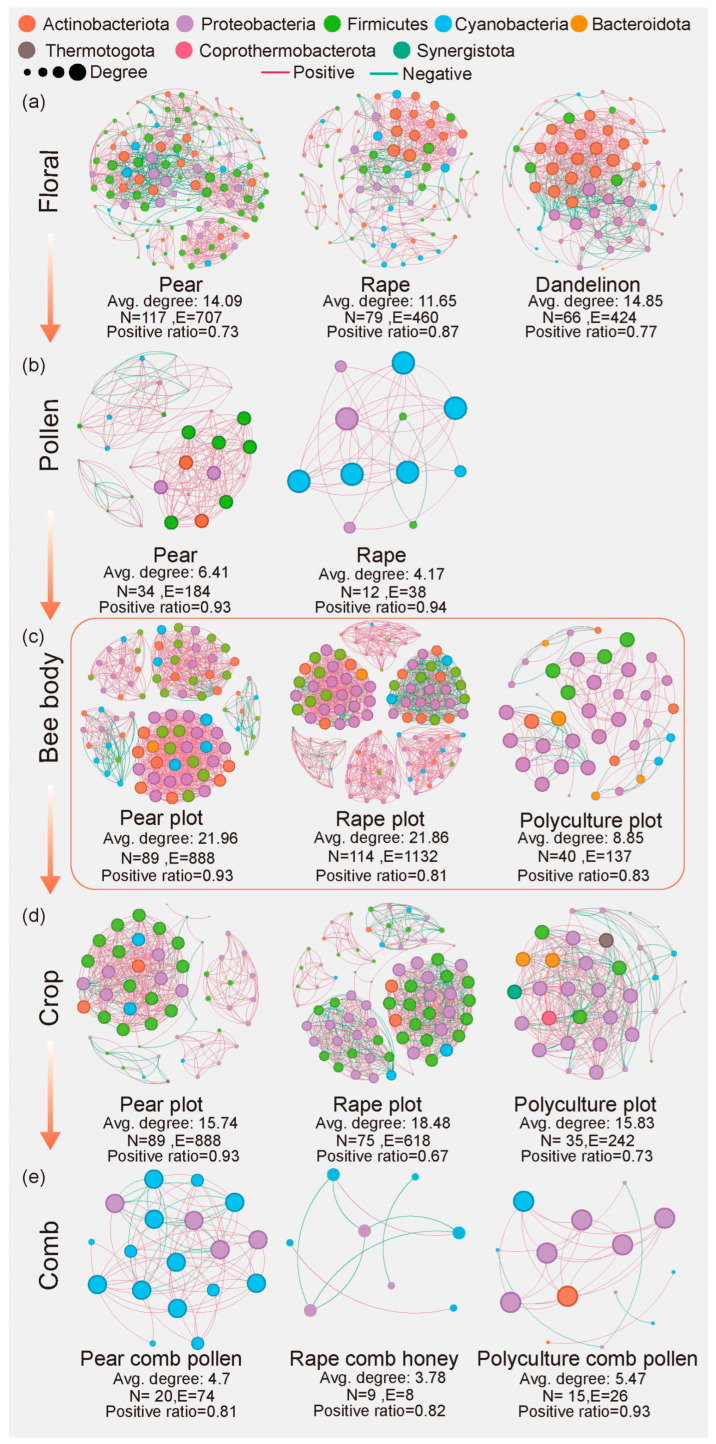
Co-occurrence network analysis of bacterial communities from floral organs to the hive. Illustrating the route of bacterial movement during the foraging process in monoculture plots (Pear, Rape) and the polyculture plot. The microbial network on the bee body surface showed the highest average degree, indicating its key role in connecting environmental, floral, and hive microbiomes. (**a**) Co-occurrence network of floral microbiomes from three different plots, with the polyculture plot only presenting the microbiome on Dandelion flowers. Co-occurrence networks of bacterial communities on pollen from anthers (**b**), the bee body surface (**c**), the crop (**d**), and comb samples (**e**) in each plot.

The co-occurrence networks reveal that the surface of honey bees (*Apis mellifera*) functions as the microbial connectivity center, demonstrating the highest average degree (pear: 21.86; rape: 21.96; Figure 3c). This indicates a tightly interconnected network of microbial communities on the bee body in monoculture environments. In the polyculture plot, however, the avg. degree of bee body microbial networks decreased significantly (avg. degree = 8.85; Figure 3c), likely influenced by bees foraging on multiple plant species and mixing diverse floral microbial communities. This trend suggests that monoculture environments promote microbial cohesion on bee surfaces, whereas polyculture environments break this cohesion due to microbiome mixing.

Among the different plant-associated niches, the bacterial communities on flower surfaces showed significantly higher connectivity (avg. degree = 19.63; Figure 3a) compared to pollen (avg. degree = 5.47–15.83; Figure 3b) and nectar or honey (e.g., pear flower honey, avg. degree = 12.14; Figure S4c). These results highlight flower surfaces as hotspots for microbial interactions, potentially supporting microbial exchange between floral visitors like bees. The reduced connectivity in pollen and nectar communities suggests compartmentalization of microbial activity in these substrates.

The microbial network in honeybee crops exhibited relatively stable connectivity (avg. degree = 18.48; Figure 3d), dominated by three bacterial phyla: Proteobacteria, Firmicutes, and Actinobacteriota. In hive matrices (comb pollen and comb honey), connectivity significantly dropped (avg. degree as low as 3.78 in rape comb honey), with Proteobacteria and Cyanobacteria showing the strongest interactions. This shift suggests the selective filtering of microbial communities during the transition from bee crops to hive environments.

Environmental microbiomes displayed the highest microbial diversity and richness, as confirmed by the Shannon diversity and Chao1 indices (Figure S2b). Floral microbiomes varied significantly across different plants and cultivation plots (Figure S2c), with flowers from monoculture pear and rape plots forming distinct clusters compared to those in the polyculture plot. These compositional changes were also evident in the network properties analyzed across plots (Figure S4a). For instance, *Orychophragmus violaceus*, a unique species in the polyculture plot, displayed a highly connected floral microbiome (Figure S4b), likely influencing community structures in the mixed planting environment.

### 3.3. Bees Carry the Microbial Signature of the Cropping System

**Figure 4:**
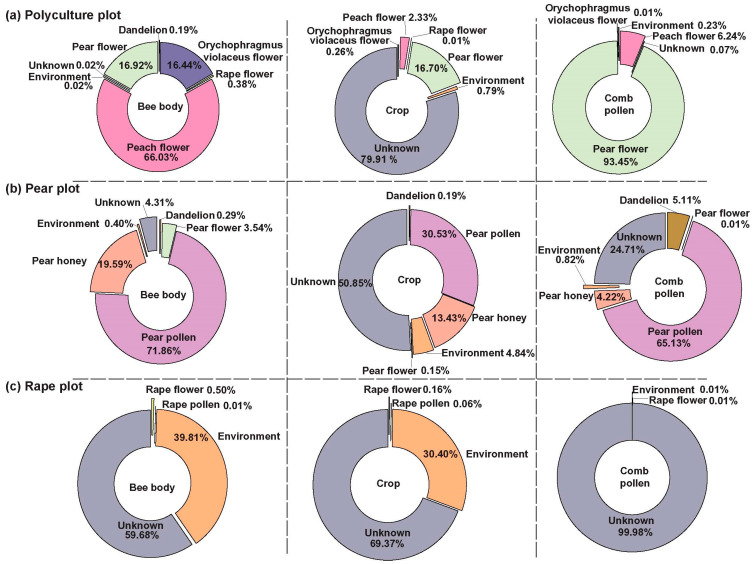
The source model of the bee body surface, honey crop, and comb pollen microbiome in the polyculture plot (**a**), pear monoculture plot (**b**), and rape monoculture plot (**c**), constructed by fast expectation–maximization microbial source tracking (FEAST), revealed the potential sources of bacterial communities. The microbial source composition of the crop remained relatively stable, while the microbial sources carried by bees in polyculture systems were more diversified, which may enhance their adaptability to environmental changes. The pie charts show the proportional composition of microbial sources for each sample type. Different colored sections in the pie charts represent the contributions of distinct microbial sources.

In the polyculture plot, the microbiota on the bee body surface primarily originated from peach flowers (66.03%), with additional contributions from pear flowers (13.92%) and *Orychophragmus violaceus* flowers (16.44%). In comparison, a large proportion of microbiota in the honey crop was of unknown origin (79.91%), while most of the microbiota in comb pollen was derived from pear pollen (93.45%), indicating a strong influence of surrounding vegetation (Figure 4a). In the pear monoculture plot, the microbiota on the bee body surface was mainly associated with pear-related sources, including pear pollen (71.86%), pear honey (19.59%), and pear flowers (3.54%). The honey crop microbiota included a significant proportion of unknown origin (50.85%), as well as substantial contributions from pear pollen (30.53%) and pear honey (13.43%). The microbiota in comb pollen was predominantly derived from pear pollen (65.13%), demonstrating the concentrated influence of a single crop planting (Figure 4b). In the rape monoculture plot, the microbiota on the bee body surface was mainly derived from unknown sources (59.68%) and environmental microbes (39.81%). The honey crop microbiota contained a high proportion of unknown microbes (69.37%), followed by environmental microbes (30.40%). For comb pollen, the microbiota was almost entirely of unknown origin (99.98%) (Figure 4c).

The microbiota on the bee body surface is strongly influenced by the pollination environment in different planting plots. In contrast, the honey crop microbiota seems to maintain a specific composition, with a large proportion (50.85–79.91%) of unknown origin. In the rape monoculture plot, the very high proportion of microbiota from unknown sources (59.68–99.98%; Figure 4c) can be attributed to several factors. These include the limited vegetation sampling in monoculture plots, which disproportionately excludes potential alternative sources; the uncharacterized microbiomes of local nectar resources that bees utilize for foraging; and the likely off-plot foraging activities of bees, given the restricted diversity within monoculture plots.

## 4. Discussion

The bee microbiome (BeeBiome) is closely associated with bee health [9,13,14], playing a crucial role in shaping microbial dynamics within agricultural ecosystems. However, the mechanisms underlying its distribution and interactions across various agricultural components remain insufficiently explored [24,25]. This study highlights the bee body surface as a key hub for microbial exchange, emphasizing its role in facilitating interactions among diverse microbial communities. Additionally, we uncover distinct compositional characteristics of crop microbiomes, shedding light on the central role of bees as indispensable nodes in agricultural microbial networks.

### 4.1. Floral Microbiomes of Different Nectar Plants Remain Relatively Stable

Environmental microbial communities, influenced by surrounding factors, do not exhibit significant clustering patterns on the NMDS plot but rather appear more dispersed, reflecting the high diversity and structural complexity of aerosol-associated microbiomes. In contrast, the floral microbiomes of different plant species (or cultivation sites) show significant differences, characterized by the independence and differentiation of microbial communities (Figure 2b and Figure S2b). Despite visiting multiple plants, bees and other pollinators do not significantly mix or homogenize floral microbiomes (Figure 2b). This indicates that floral microbiomes exhibit strong host specificity or environmental adaptability, with the floral microhabitat playing a key role in shaping their composition [26]. Consequently, bee-mediated dispersal has limited influence on forming mixed microbial communities [27]. Supporting this, studies have shown that foraging pollinators have minimal impact on the bacterial communities of strawberry flowers in open fields [28]. This finding supports the “floral niche model,” which suggests that the chemical composition of nectar, the microstructure of petal surfaces, plant volatile signals, and the nutritional profile of pollen collectively act as selective filters, allowing only specific bacterial strains to colonize and thereby driving community differentiation [26,29].

This illustrates that plant floral niches provide microbes with a relatively stable and resource-rich microenvironment, resembling “ecological islands” that promote independent community evolution and the maintenance of diversity. In contrast, the atmospheric environment acts more like a “continuum,” dominated by constant input and washing processes, making it difficult to establish stable community boundaries. This comparison not only highlights the critical role of habitat types in shaping microbial β-diversity patterns but also emphasizes the need to focus on meteorological drivers at the regional scale when monitoring airborne plant pathogens. Meanwhile, in managing beneficial crop-associated microbes, efforts should prioritize precise matching between host varieties and floral microenvironments.

### 4.2. Bees as a Keystone in Microbial Communication Within Agricultural Systems

Bees play an essential role as a “bridge” in microbial communication within agricultural systems [27,30]. Our study confirms that microbial communities associated with their body surface, crop, and internal and external habitats exhibit remarkable diversity and complex connectivity. These microbial communities are not only derived from the bees themselves but are also strongly influenced by environmental factors and cropping patterns [28,31,32]. As a “transit hub” for microbes in ecosystems, the bee-associated microbiome displays high connectivity (Figure 3c), facilitating the transfer of microbes between the hive, pollinated plants, and the surrounding environment.

As a key intermediate hub for microbial exchange, the bee-associated microbiome exhibits high connectivity across different cultivated plots. Specifically, the surface of honey bees showed the highest average degree among niches, reaching 21.86–21.96 in pear and rape monoculture plots (Figure 3c), forming a tightly interconnected microbial network in monoculture environments. This connectivity underscores the central role of bees in facilitating extensive microbial interactions among hives, pollinated plants, and the surrounding environment [31,33]. Notably, planting systems shape microbial network dynamics: monoculture environments reinforce microbial cohesion through restricted plant diversity, while polyculture systems introduce diverse floral sources, fragmenting microbial networks and reducing cohesiveness on bee surfaces. These findings highlight the dual influence of agricultural practices and bee behavior on microbial network properties.

From the perspective of microbial network co-occurrence connectivity, the bee body surface, rather than flower-associated microbiomes, represents the critical hub for microbial integration and exchange. Our results demonstrate that the microbial networks on bee surfaces exhibit the highest degrees of cohesiveness and connectivity, far exceeding those of floral microbiomes. This highlights the unparalleled role of bees in serving as a microbial communication hub, where their body surfaces act as central nodes for microbial exchange and interaction [34,35]. The discovery emphasizes the ecological value of bees in facilitating microbial connectivity and information flow across different ecological compartments, making them indispensable in maintaining the integrity and functionality of microbial networks in agricultural ecosystems. These findings emphasize the significance of bees not only as microbial transporters but also as keystone species in sustaining microbial diversity and ecological stability in pollination networks [36,37]. Moreover, the results obtained demonstrate that increasing plant diversity in cropping systems plays a crucial role in maintaining microbial connectivity in ecosystems. Optimizing agricultural practices by balancing crop productivity with ecological diversity is essential to maintaining bee health and strengthening their role as a microbial exchange hub.

### 4.3. Variability in Microbial Sources and the Unique Composition of Crop Microbiomes

The data presented herein serve to reinforce the view that the floral landscape within a colony’s foraging radius exerts a significant influence on the surface microbiome of the honeybee, as well as that of its stored comb pollen. The imposition of a uniform microbial signature on bee cuticles and pollen reserves was observed to occur as a consequence of a monoculture of pears. In contrast, a polyculture of pear, peach, Orychophragmus, and dandelion blooms was found to result in the weaving of a correspondingly mixed microbial tapestry on bees and in their pollen stores (Figure 4a,b). Ecologically, the surface microbiome acts as a real-time floral ledger that mirrors landscape botanic composition [38], while monoculture-driven loss of this diversity may strip bees of microbial allies vital for nutrition and pathogen defense [10,38]. While greater microbial diversity in cropping systems can enhance ecological stability, it may also increase the likelihood of harboring microorganisms that could negatively affect bee health [39]. Management practices such as maintaining floral diversity, crop rotation, and targeted microbial interventions can help promote beneficial communities while limiting potential pathogens. Future work should incorporate targeted pathogen screening and monitoring of potentially harmful taxa to better evaluate and mitigate risks to bee health in diverse agricultural systems.

Interestingly, honeybee crop microbiota exhibited a unique composition that was only partially influenced by environmental microbial sources. Across all cultivation plots, the proportion of microbial taxa of unknown origin within the crop microbiota was notably high, ranging from 50.85% in pear monoculture plots to as high as 79.91% in polyculture systems (Figure 4a–c). This indicates that, unlike the bee body surface, the crop microbiota may comprise not only environmental microbes but also a specialized and distinct microbial community potentially shaped by physiological and biochemical conditions unique to the honey bee crop. Warren et al. further support our conclusion by showing that the honeybee crop maintains a distinct, season-stable microbiome, confirming that crop-associated bacteria are conserved rather than transient environmental contaminants [24]. The crop microbiota differs markedly from the nectar microbiota, indicating that the crop acts as a selective enrichment or filtration step that eliminates a subset of environmental bacteria and establishes a community composition distinct from that of floral nectar [24,39,40]. Prior research has suggested that bees may actively modify the nectar they collect through enzymatic processes, such as the secretion of enzymes from salivary glands [41]. These modifications potentially result in a microbial community that is distinct from the floral microbiomes and influences microbial composition from the very beginning of the pollen and nectar collection phase.

Notably, the analysis revealed that a large proportion of microbial taxa in rape plot samples (specifically from bee body surfaces, honey crop microbiota, and comb pollen) could not be attributed to prior environmental sources or the flowers identified in this study (Figure 4c). These “unknown” sources accounted for 59.68–99.98% of sequences in the rape monoculture, likely reflecting limited sampling coverage. First, local rape cultivars produced minimal nectar, precluding the collection of rape-floral nectar; as nectar microbiota represent a key potential source of rape-associated microbes, their absence directly inflated the proportion of unassigned reads. Second, the potential influence of nearby nectariferous plants outside the designated plots must be considered, particularly in monoculture systems where bees forage beyond the cultivated area. Future studies should expand vegetation sampling to achieve a more comprehensive understanding of microbial sources available to foraging bees in agricultural landscapes.

## 5. Conclusions

This study provides evidence that cropping systems shape the structure and connectivity of bee-associated microbiomes, primarily through differences in the floral landscapes accessible to foraging bees. The microbiota on bee body surfaces and in comb-stored pollen closely reflected the surrounding vegetation, with monoculture plots producing more uniform microbial signatures and polyculture plots generating more heterogeneous mixtures. In contrast, the honey crop microbiota maintained a distinctive and partially unknown composition across all plots, indicating selective processes associated with bee physiology. Network analyses further revealed that monoculture systems reinforced tighter microbial cohesion on bee surfaces, whereas polyculture systems introduced more fragmented interaction patterns. Together, these findings highlight how planting regimes influence microbial assemblages linked to honeybees while also underscoring the need for future studies to examine the functional implications of these microbiome shifts for bee health and agricultural ecosystems.

## Data Availability

The original contributions presented in this study are included in this article/Supplementary Materials. Further inquiries can be directed to the corresponding authors.

## Supplementary Materials

The following supporting information can be downloaded at: https://www.mdpi.com/article/10.3390/insects17010053/s1, Figure S1: The study plots are located in Yuncheng City, Shanxi Province, China, showing the precise sampling sites within this region; Figure S2: Microbial diversity (Shannon diversity and Chao1 richness) in environmental, flower, crop, and bee-comb microbiomes across polyculture, pear monoculture, and rape monoculture plots. (a) Shannon diversity and Chao1 richness of the crop microbiome. (b) Shannon diversity and Chao1 richness of the environmental microbiome. (c) Shannon diversity and Chao1 richness of the flower microbiome associated with *Orychophragmus violaceus*, dandelion, peach, pear, and rape. (d) Shannon diversity and Chao1 richness of the bee-comb microbiome, including bee body, comb, and flower samples from polyculture, pear, and rape plots. Different letters indicate significant differences (*p* < 0.05); Figure S3: Venn diagram showing the shared and unique ASVs representing the floral microbiomes from five different plant species; Figure S4: (a) Co-occurrence network of microbial communities from mixed environmental samples across monoculture plots (Pear, Rape) and the polyculture plot. (b) Co-occurrence network of bacterial communities on *Orychophragmus violaceus* flowers in the polyculture plot. (c) Co-occurrence network of bacterial communities in pear flower honey, derived from multiple open pear flowers collected from the monoculture pear plot and mixed for microbial analysis.
