# Supplementary material for: The Honey Bee Body Surface as a Microbial Hub: Connectivity Shaped by Monoculture vs. Polyculture Farming
Source: Insects. 2026 Jan 1;17(1):53. doi: 10.3390/insects17010053 (PMC12842557; doi:10.3390/insects17010053)
Supplement: Supplementary file 1 [file insects-17-00053-s001.zip › insects-4029242-supplementary.pdf]

The Honey Bee Body Surface as a Microbial Hub: Connectivity Shaped by Monoculture vs. Polyculture Farming

Baobei Guo [1,2,*], Xueyan Yi[3], Qihang Sun[3], Ke Sun[3], Lina Guo[3], Yuan Guo[4,*]

1 Pomology Institute, Shanxi Agricultural University, Taiyuan, 030031, China; guobaobei@sxau.edu.cn

2 Shanxi Key Laboratory of Fruit Germplasm Innovation and Utilization, 030031 Taiyuan, China

3 College of Animal Science, Shanxi Agricultural University, Jinzhong 030801, China; 18783584670@163.com (X.Y.); qihangsun1999@163.com (Q.S.); 13239896557@163.com (K.S.); linaguo@126.com (L.G.)

4 College of Horticulture, Shanxi Agricultural University, Taiyuan 030031, China; yysgy3@sxau.edu.cn

* Correspondence: (B.G.), E-mail: guobaobei@sxau.edu.cn; Tel.: +86-0351-7639482; ORCID: https://orcid.org/0000-0001-6241-678X; (Y.G.), E-mail: yysgy3@sxau.edu.cn; Tel.: +86-0351-7639482

Supplementary methods

Determination of the proportion of nectariferous plants in polyculture plot

The proportions of plant species in the polyculture plot were determined based on nectariferous plants blooming concurrently within a 3-km radius of the apiary. By observing and documenting the foraging behaviors of honeybees, the attractiveness of each plant species to pollinators was systematically evaluated. Using simple field measurements, the final planting ratios were established as follows: pear (50%), rape (25%), peach (10%), *Orychophragmus violaceus* (9%), dandelion (3%), and supplementary nectar-providing taxa (3%), thereby ensuring the diversity of nectar plants within the polyculture plot. All experimental forage crops were in bloom during the study period. However, it should be noted that by the time samples were collected from the polyculture plot, peach blossoms had entered their end-of-flowering stage and only a small portion of peach flowers remained open.

Detailed Sample Collection Procedures

On March 29, 2022, microbial samples were systematically collected after three consecutive days of normal honey bee foraging activity, adhering to predefined experimental parameters. In the pear monoculture plot, floral microbial communities were sampled from 10 randomly selected pear blossoms at full bloom. The blossoms were randomized into 50 mL sterile tubes, subjected to three cycles of 30 mL phosphate-buffered saline (PBS, pH 7.0) washes (1 min vortexing at 2,500 rpm, 1 min ultrasonic sonication at 40 kHz), with eluates filtered through 0.22 μm nitrocellulose membranes (EHK®, China) (five biological replicates). Pollen microbiota were similarly ex-tracted by PBS elution and filtration (1 g fresh pollen per sample, five replicates), nectar was collected using 0.5ml sterile syringes, which were used to draw nectar into 1.5 mL EP tubes (1 mL per replicate, five replicates), and Concurrently, foraging bees captured at hive entrances were subjected to sequential surface decontamination using phos-phate-buffered saline (PBS, pH 7.0) through three cycles

of vortex oscillation (2,500 rpm, 1 min) and ultrasonic sonication (40 kHz, 1 min), with resultant filtrates concentrated on 0.22 μm membranes. Post-wash bees were aseptically dissected to harvest crops (bee stomach, n=5 per replicate) for microbiome analysis. Hive matrices, including stored pollen and honey, were cored from central combs using sterilized boring tools. The collected samples were transferred into 1.5 mL EP tubes, with sampling repeated 5 times to ensure consistency. Environmental bioaerosols were captured via a 90 mm qualitative filter paper (BKMAMLAB®, China) mounted at 1.5 m elevation for 120 hours of exposure. After collection, the filter papers were eluted with PBS following the floral microbiome sampling protocol, with five replicates processed.

Pollen samples were collected from pear flowers in both the pear monoculture and the polyculture plot, as well as from rape flowers in the rape monoculture plot. Using sterile forceps, dehiscent anthers from pear or rape flowers were carefully transferred into 2.0 mL EP tubes. Each tube contained 1 g of anthers, with five biological replicates collected per sample. The microbial communities associated with the collected pollen were analyzed following established protocols for sampling flower-surface microbiomes. To sample the collected pollen microbiome, each pollen sample was placed into a 50 mL sterile centrifuge tube and subjected to three sequential washes using 30 mL phosphate-buffered saline (PBS, pH 7.0). Each wash consisted of 1 minute of vortexing at 2,500 rpm followed by 1 minute of ultrasonic sonication at 40 kHz. The resulting eluates were filtered through 0.22 μm nitrocellulose membranes (EHK®, China). Five biological replicates were processed for each sample.

DNA Extraction, PCR Amplification, and Bioinformatic Processing

Microbial DNA was isolated from floral microbial communities, pollen microbiota, nectar, honeybee integument eluates, aseptically dissected crop contents, stored pollen/honey cores (from hive matrices), and environmental bioaerosol-filtered samples. Extraction was performed using the FastDNA™ Spin Kit (MP Biomedicals,

Southern California, USA) following the manufacturer's protocol. The PCR conditions were as follows: initial denaturation at 95 °C for 3 minutes, followed by 27 cycles of denaturation at 95 °C for 30 seconds, annealing at 55 °C for 30 seconds, and extension at 72 °C for 45 seconds, with a final extension at 72 °C for 10 minutes. The 20 μL PCR mixture contained 4 μL 5 × TransStart FastPfu buffer, 2 μL 2.5 mM dNTPs, 0.8 μL forward primer (5 μM), 0.8 μL reverse primer (5 μM), 0.4 μL TransStart FastPfu DNA Polymerase, 10 ng template DNA, and ddH_2O up to 20 μL. Each sample was tested in triplicate. DNA concentration and purity were assessed using a NanoDrop 2000 spectrophotometer, and structural integrity was examined by electrophoresis on 1% agarose gels.

Bacterial community profiling targeted the V3–V4 hypervariable region of the 16S rRNA gene. PCR amplification employed primers 338F (5′-ACTCCTACGGGAGGCAGCAG-3′) and 806R (5′-GGACTACHVGGGTWTCTAAT-3′) on a T100 Thermal Cycler. The resulting amplicons were purified using a dedicated PCR cleanup kit and quantified with a Qubit 4.0 fluorometer to ensure equimolar pooling for library construction. Sequencing was performed on an Illumina MiSeq platform using a paired-end 250 bp strategy, generating high-quality reads suitable for amplicon-based microbial community reconstruction.

Bioinformatic processing followed a standard ASV-based pipeline. Raw sequences were first subjected to quality trimming and adapter removal using fastp, after which high-quality paired reads were merged through FLASH. Amplicon sequence variants were inferred using the DADA2 algorithm, which incorporates error modeling and chimera removal to generate exact biological sequences. Taxonomic annotation was performed in Qiime2 against the SILVA 16S rRNA reference database (version 138), enabling classification from phylum to genus levels. To account for variation in ribosomal operon copy number among taxa, ASV abundances were normalized using information from the rnDB database. This comprehensive workflow ensured accurate,

resolution-enhanced characterization of microbial communities across diverse floral, bee-associated, hive, and environmental sample types.

statistical analysis

The co-occurrence network analysis was performed using the R packages ‘psych’ and ‘reshape2’ using the Spearman correlation matrix and visualized in Gephi (https://gephi.org/). Statistically significant ($P<0.05$) and strong relationships (Spearman's $r > 0.6$ or $r < -0.6$) were observed. The bee body exhibited the highest average network degree among all niches, indicating its central role in fostering interactions among microbial taxa. High degree reflects dense connectivity, suggesting the bee body serves as a hub for microbial exchange, integrating diverse microbes from floral surfaces, nectar, pollen, and the surrounding environment. This network connectivity highlights the bee body as a critical platform for microbial interaction and convergence.

Supplementary figure

Figure S1

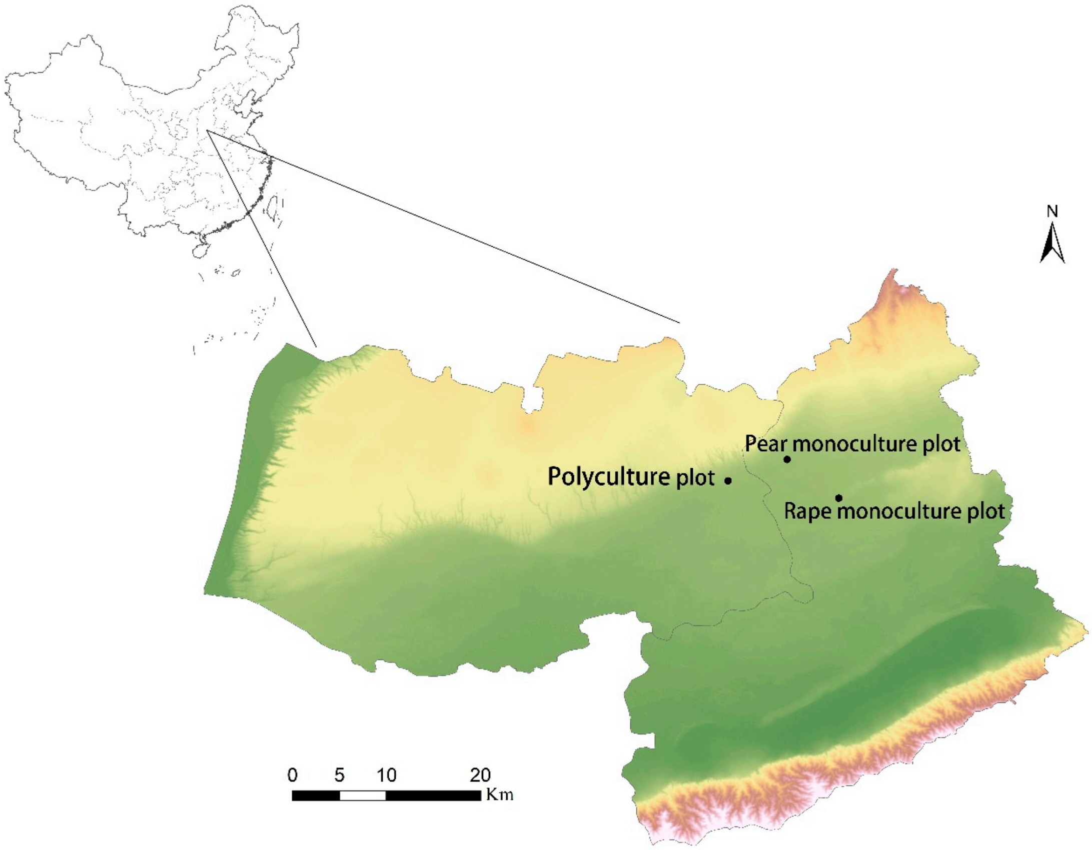

Figure S1. The study plots are located in Yuncheng City, Shanxi Province, China, showing the precise sampling sites within this region.

Figure S2

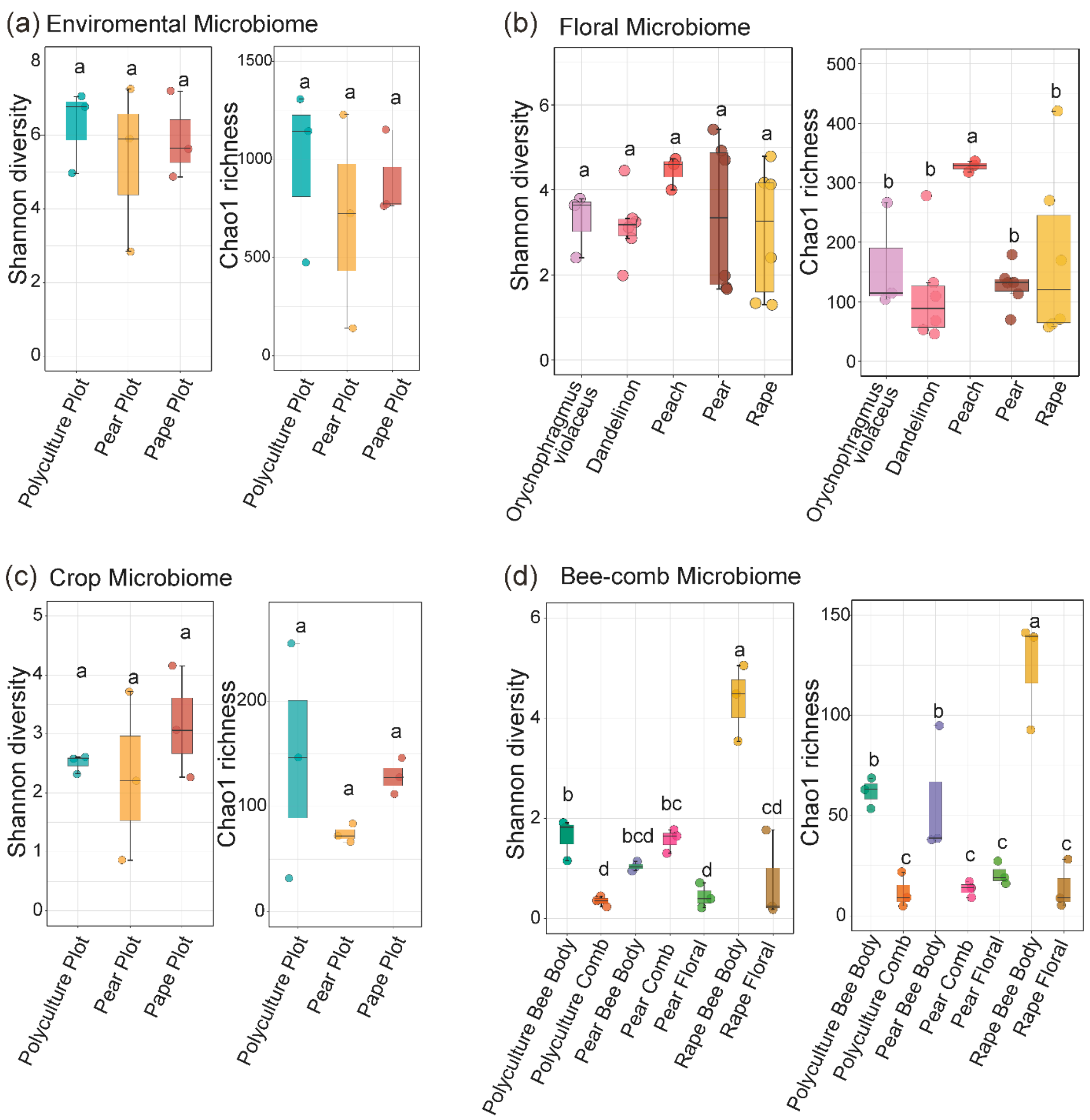

Figure S2. Microbial diversity (Shannon diversity and Chao1 richness) in environmental, flower, crop, and bee-comb microbiomes across polyculture, pear monoculture, and rape monoculture plots. (a) Shannon diversity and Chao1 richness of the crop microbiome. (b) Shannon diversity and Chao1 richness of the environmental microbiome. (c) Shannon diversity and Chao1 richness of the flower microbiome associated with Orychophragmus violaceus, dandelion, peach, pear, and rape. (d) Shannon diversity and Chao1 richness of the bee-comb microbiome, including bee body, comb, and flower samples from polyculture, pear, and rape plots. Different letters indicate significant differences ($P < 0.05$).

Figure S3

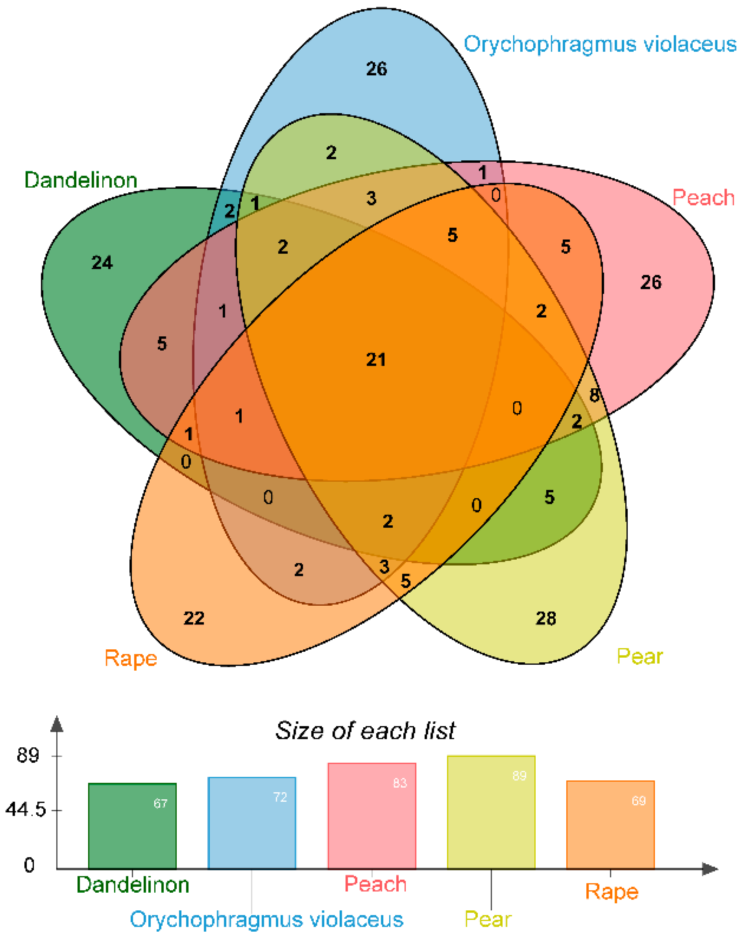

Figure S3. Venn diagram showing the shared and unique ASVs representing the floral microbiomes from five different plant species.

The Venn diagram illustrates the distribution of ASVs among the floral microbiomes of five different plants: Dandelion, Orychophragmus violaceus, Peach, Pear, and Rape. Each circle represents the set of ASVs detected in the floral microbiome of a particular plant species. Overlapping areas indicate ASVs shared among two or more plant species, while non-overlapping areas represent ASVs unique to a specific plant's floral microbiome (Figure made by jvenn from http://jvenn.toulouse.inra.fr/app/index.html).

Figure S4

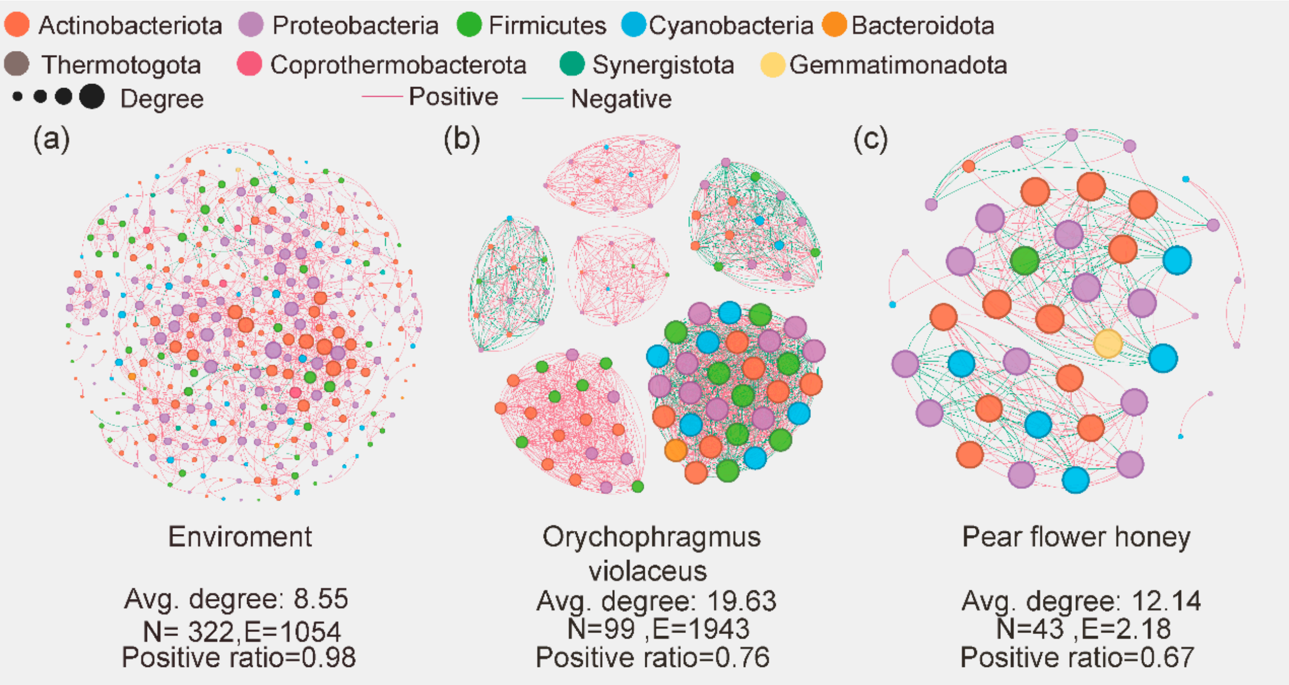

Figure S4. (a) Co-occurrence network of microbial communities from mixed environmental samples across monoculture plots (Pear, Rape) and the polyculture plot. (b) Co-occurrence network of bacterial communities on *Orychophragmus violaceus* flowers in the polyculture plot. (c) Co-occurrence network of bacterial communities in pear flower honey, derived from multiple open pear flowers collected from the monoculture pear plot and mixed for microbial analysis.
